# Public health disinformation, conflict, and disease outbreaks: a global narrative integrative review to guide new directions for health diplomacy

**DOI:** 10.1080/16549716.2025.2562380

**Published:** 2025-09-23

**Authors:** Laura E. R. Peters, Gina E. C. Charnley, Stephen Roberts, Ilan Kelman

**Affiliations:** a Department of Risk and Disaster Reduction, University College London, London, UK; b College of Earth, Ocean, and Atmospheric Sciences, Oregon State University, Corvallis, OR, USA; c Global Health Resilience Group, Earth Sciences, Barcelona Supercomputing Center – Centre Nacional de Supercomputació, Barcelona, Spain; d Department of Infectious Disease Epidemiology, School of Public Health, Imperial College London, London, UK; e Institute for Global Health, University College London, London, UK; f UiT The Arctic University of Norway, Tromsø, Norway

**Keywords:** disinformation, misinformation, health diplomacy, public health information, health conflict

## Abstract

The COVID-19 pandemic laid bare the unpreparedness of global and public health systems to respond to large-scale health crises, while simultaneously revealing the entangled nature of disinformation and poor global and public health outcomes. This research challenges the common treatment of public health disinformation – deliberately false information – as an emergent and technical threat, and instead situates it as a more systemic and nuanced challenge for global health governance to address. This article presents an integrative narrative literature review on the interlinkages between public health disinformation, conflict, and disease outbreaks, demonstrating mutually influencing connections between them. In doing so, the analysis raises critical questions around how reactive responses, such as doubling down on information authority, can paradoxically fuel the uptake of both disinformation especially amidst global trends towards increasing conflict and decreasing cooperation. In this evolving sociopolitical landscape for global health, the discussion explores the potential to harness health diplomacy to strengthen critical public engagement and deliberation. This reimagined approach to health diplomacy offers pathways to mitigate the harmful effects of disinformation rather than seeking to eliminate false information. This article contributes to deepening an understanding of this rapidly expanding topic for global and public health in two pathways. First, by investigating the root causes and impacts of public health disinformation that intersect with conflict. Second, by exploring how health diplomacy can foster cooperative global health governance through transparency and inclusion. This research offers a new direction to strengthen preparedness for future global and public health crises amidst disinformation.

## Paper Context

- **Main findings**: This review demonstrated that disinformation has long been used as a tool to advance political goals, and public health is one of the most relevant arenas where actors apply strategies to disrupt and destabilise. Measures to combat public health disinformation that overlook sociopolitical dimensions may paradoxically fuel this systemic challenge to global health.
- **Added knowledge**: This article contributes to deepening an understanding of this rapidly expanding topic for global and public health in two key ways. First, by investigating the root causes and impacts of public health disinformation that intersect with conflict. Second, by exploring how health diplomacy can foster cooperative global and public health governance through transparency and inclusion.
- **Global health impact for policy and action**: The article explored health diplomacy and how it could be leveraged to mitigate the impacts of disinformation – namely, the breakdown of systems of information and trust – through improving mechanisms for transparency and inclusion in global and public health governance.

## Background

The COVID-19 pandemic (2020–2023) demonstrated how unprepared the global and public health community was – and continues to be – to mitigate and respond to disease outbreaks [1–3]. Public health failures were seen at all stages of the emergency period of the pandemic, tightening and relaxing public health measures, and distributing life-saving resources [4]. These failures resulted in severe disruptions that affected global systems and upended lives [5], including 5.42 million COVID-19-related deaths reported. This is likely to be a gross underestimate, with 14.83 million excess deaths statistically modelled [6]. Excess deaths refer to ‘the difference in the total number of deaths in a crisis compared to those expected under normal conditions’ [7, p.5], and, for comparison, the total number of overall global deaths was estimated to be 64 million in 2020 and 67 million in 2021 [8].

Evidence has mounted that the proliferation of global streams of false information exacerbated public health challenges and worsened population and public health outcomes during the COVID-19 pandemic [9–14]. Disinformation refers to intentionally fabricated information, while misinformation refers to false information produced and shared without the intent to deceive or mislead. While not a new or geographically isolated phenomenon, for example, the topic of disinformation is explored in ancient Greece [15] and has long existed for diseases like smallpox [16] and polio [17] – the COVID-19 pandemic marked an acceleration of public health disinformation around the disease, vaccines, and other public health measures [18,19]. In a health crisis that called for disparate actors and populations to act in concert, disinformation was leveraged to deepen divisions and disconnections between the people and systems that ‘depend on, influence, and interact with each other’ for public health [20, p.1).

Public health disinformation has been recognised as a global health threat [21], and concerns have centred on the technical aspects, including disinformation campaigns powered by artificial intelligence and targeted via enhanced analytics. The ‘disinformation age,’ including its extensions to public health, has been facilitated by technologies and data that empower anyone with access to swiftly generate and widely disseminate information regardless of its veracity [22]. While important, these considerations do not grapple with the underlying offline sociopolitical dimensions that make global and public health disinformation such a formidable problem to address, especially amidst global trends of escalating conflicts and deteriorating peacefulness [23].

The integrative narrative literature review presented in this article explores the complex relationships and patterned dynamics between public health disinformation, conflict, and disease outbreaks, without presuming or seeking to establish direct causality. Instead, the review investigates the entangled nature of these challenges to consider how global and public health governance can develop more holistic solutions. Based on the review, the discussion argues that critical public engagement and constructive deliberation are needed to be better prepared for future pandemics in the evolving sociopolitical landscape for global and public health. The article concludes with a call to action for health diplomacy along with actionable recommendations.

## The research process

This section clarifies operational definitions before detailing the research process.

### Operational definitions

Misinformation is incorrect material, while disinformation, as a subset, is deliberately incorrect material with the intention to mislead [24]. This straightforward distinction comes from English dictionaries, since deep theoretical explorations and developments of these ideas, and variations such as malinformation and fake information, are not well-developed [25,26]. A systematic review of public health literature using terms related to misinformation and disinformation found general agreement that health disinformation is ‘the intentional dissemination of false information’ [27, p.6] but without specifying the relationship to public health. The present research thus offers an operational definition of public health disinformation as the intentional dissemination of false information related to health that may or may not influence public health outcomes.

Conflict refers to the real or perceived incompatibilities of goals, interests, or values between two or more parties [28]. Conflicts are often related to complex and systemic issues, as distinguished from disputes that are over specific issues [29]. Conflict holds the potential to be constructive and lead to improved relationships, mechanisms, and outcomes [28]. However, without the presence of robust societal conflict resolution mechanisms that are effective, sustainable, and acceptable to all parties, conflict can manifest in multiple forms including armed conflict and combat, organised and unorganised political violence, organised crime and terrorism, state suppression, and systemic racism and structural inequalities [30] – all of which are considered in the present research.

Public health refers to ‘the science and art of preventing disease, prolonging life and promoting health through organised efforts of society’ [31, p.1], and global health is defined as ‘collaborative trans-national research and action for promoting health for all’ [32, p.1] Some have argued against distinguishing between public and global health, with Fried et al. [33, p.536] writing, ‘Public health is global health for the public good.’ In applied settings, public health agencies are typically mandated to save lives by providing guidance to political leaders and the public, as well as by formulating and administering interventions to prevent and control disease spread in their political jurisdictions. Global health agencies historically work closely with state governments and elected and appointed health officials through cooperation to advance public health and improve health equity within and across borders.

### The research process

The purpose of an integrative narrative literature review is to offer a representative description of a topic intellectually grounded in different disciplinary conversations and advancements, conduct critical analysis on broad questions, and generate new insights including through conceptual theorising [34–38]. The present research is thorough and representative but, in line with guidelines on narrative literature reviews, does not follow a systematic methodology to review all available literature on related topics due to the wide breadth of the subject and disciplinary perspectives on it. The restrictive rules of a systematic review would impede the development of the wider narrative from multiple debates and literatures [39], but in not establishing and describing specific inclusion and exclusion criteria, the limitations of a narrative review are that they are more subjective and less reproducible [38,39]. Nevertheless, narrative literature reviews offer distinct benefits when compared with systematic reviews such as meta-analyses and traditional systematic reviews [38].

The guiding research question was to explore the complex relationships and patterned dynamics of public health disinformation, conflict, and disease outbreaks. The research was undertaken based on the authors’ interdisciplinary expertise in global and public health, global health security and big data, health emergencies and disasters including applications for risk reduction, and peace and conflict studies. The authors conducted research on the following complex relationships: disinformation and conflict, public health disinformation and conflict, and conflict and disease outbreaks. The authors expanded on their knowledge of the literature by conducting searches in academic research databases, including seeking mainstream, alternative, and dissenting studies and perspectives, and seeking contemporary examples and emergent trends from news outlets and gray literature.

To capture the evolving debates on long-standing topics, the authors included both recent publications and earlier works across conceptual and empirical research. The authors sourced material from and about diverse global geographies. Special care was taken to seek information about a broad and diverse spectrum of conflict-affected contexts, including as identified in the Global Peace Index [23], rather than focusing just on high-intensity armed conflict zones. This was done to avoid reinforcing reductionist stereotypes about certain regions and implying that these challenges are theirs alone, and to strengthen the analytical rigour by identifying systemic patterns rather than anomalies specific to these contexts.

The authors conducted the research iteratively: each author conducted thematically overlapping searches and wrote summary reviews starting from January 2024; the authors met virtually on Zoom to discuss findings and shape the integrative narrative in March and April 2024; and the authors conducted additional targeted searches of the literature, wrote sections of text, and reviewed the manuscript in a shared document from May 2024 through January 2025. The lead author compiled the final manuscript text from February through June 2025 using the written summaries and detailed notes from team discussions, and all authors provided feedback on and approved the final manuscript.

## Intersecting fault lines for global and public health

This section presents the integrated narrative review. The first subsection describes the public health information ecosystem and its underlying politics to offer context for the relevance of conflict and its diverse manifestations on the topic. The second subsection explores the connections between disinformation and conflict situated in the fields of conflict and military studies that have long studied the topic of disinformation outside the context of public health, and which provide a baseline understanding of the origins and impacts of disinformation. The third subsection explores disinformation and public health violence, from a review of literature from the subfield of public health that focuses on conflict-affected settings.

### The politics of global and public health information

Global and public health depend on high-quality and real-time public health information, which is not merely discovered or calculated, but is also socially created. Public health information includes any data and knowledge used to support and advance health within and across political boundaries. The social creation of public health information refers to the process by which diverse actors collect, synthesise, and share data; interpret data into meaningful information; use the subsequent knowledge to inform and coordinate strategic decisions; and translate decisions into collective actions and behaviours [40]. The public health information ecosystem describes the connectivity between people and systems as they produce and share this socially created information within and across political borders. The global and public health information ecosystem is imbued with politics and ideologies around public health, including determining what variables and outcomes matter and the extent to which they should be monitored and intervened upon by authorities [40,41].

The health information ecosystem is put under strain during disease outbreaks and pandemics. Disease outbreaks, and especially those associated with emergent viruses and microbes, are characterised by rapid developments and uncertainties, and information may become quickly outdated or proven ineffective. Approximately 75% of a nationally representative sample of US adults in April 2020 reported being exposed to conflicting public health information even at that early stage of the COVID-19 pandemic [42]. Conflicting health information can lead to confusion and anxiety, impact public understanding and perceptions of risks, undermine engagement with unrelated health behaviours that have high scientific consensus, and even result in resistance to health research and recommendations more broadly [43,44]. Automated social media algorithms exacerbate polarised information bubbles by channelling conflicting health information based on personal preferences, and high levels of polarisation are linked to increased sharing of false health information [45].

Conflicting public health information may become even more divisive when considering that public health guidelines do not always lead to improved outcomes for particular people or groups, in part owing to health risk trade-offs that might not be communicated or addressed. For example, complying with physical distancing (i.e. social distancing), quarantines, and other isolation strategies helped to minimise disease transmission but augmented mental health risks [46] and put some at higher risk of interpersonal and domestic violence [47]. Further, public health information is sometimes differentiated depending on the socioeconomic and political standing. While some workers were privileged enough to continue earning wages by working from home [48], workers deemed ‘essential’ – most commonly in sectors such as healthcare, emergency services, food, water, transport, and energy – continued in close proximity with the public and coworkers to facilitate the exchange of essential goods and services. They did so by risking themselves and their families to exposure, often without adequate protective equipment due to both limited global availability and poorly managed supply chains [49]. Public health disparities are seen consistently through the disproportionate impacts suffered by those who are already structurally marginalised [50], exemplified by global vaccine inequity [51], due to lack of access to and ownership of resources and lack of inclusion in decision-making processes [52]. Given the injustices embedded within the public health system, many treat ad hoc public health guidance with suspicion during emergencies, especially where there is already low trust in authority [53,54].

Conflicting public health information, though potentially causing harm, is not necessarily disinformation, which is distinguished by the intent to deceive. Some health disinformation is motivated purely by financial gain, such as selling fraudulent or poor-quality products or services [55], while other strands of disinformation are generated by actors seeking to weaponise public health information for political influence and gain. Other disinformation may exist at the political and economic crossroads. In a high-profile example, the United States (US) President Donald Trump announced the country’s withdrawal from the World Health Organization (WHO) purportedly driven in part by the Administration’s desire to grow the country’s digital and artificial health industry with the potential to generate astronomic profits [56]. The country’s departure from the WHO enables ‘platforms run by US technology companies to spread misinformation and disinformation across borders’ motivated by commercial rather than public good [56, p.444].

While the US leads the world with the highest number of digital health companies, it is followed by India, the United Kingdom (UK), China, and Germany all which have a strong presence in the digital health sector [57]. The dark side of the increasing digital health sector’s foothold includes its potential to ‘expand state surveillance, the risk of malicious targeting, numerous challenges linked to the management of partnerships with powerful private companies, and the risks of scaling up digital interventions for which scientific evidence is weak’ [58, p.42]. New technologies and sectors, in the absence of robust governance, have shaped fertile ground for public health disinformation to proliferate.

### Disinformation and conflict

Disinformation, including propaganda and fabricated rumours, has been used to advance political goals and agendas around the world throughout history [59–61]. Disinformation is known to be used as a political weapon to control the flow of information (i.e. information warfare) [62,63]; influence people’s beliefs and behaviours (i.e. cognitive warfare) [64,65]; and recruit people to participate in political violence [66]. Disinformation can be wielded in a single instance, a campaign of multiple instances, or a longer-term operation [67]. Prominent examples of disinformation being used as a political tool in conflicts are:

- Russia’s state-sponsored disinformation campaign in 2016, which contributed to stoking race tensions in the US [68]; and
- Disinformation about Iraq’s weapons of mass destruction and involvement in the September 11th, 2001 terrorist attacks, used as a pretext for a US-led coalition to invade Iraq in 2003 [66,69].

In conflict-affected contexts, there is often not a unified trusted source of information, and unverified information may be the only source of information when people lose access to or trust in formal information sources due to armed conflict [70]. Despite this, people living in conflict zones may be less likely to believe false information due to their access to first-hand knowledge and their high motivation to understand the reality of the situation to stay safe [71]. At the same time, conflict can restrict direct and virtual access to the front lines of conflict, making these more distant populations more susceptible to false information that they cannot verify [72]. This points to the possibility that informal channels of local information may be simultaneously the most and the least accurate, especially in situations of high volatility and uncertainty and in the absence of the capacity to triangulate with other sources.

Disinformation may be less likely to create new conflict than reinforce and exacerbate existing conflict, reflecting a relationship of positive correlation rather than causation. This is in part because people are more likely to believe false information that aligns with their pre-existing biases, fears, and worldviews. Disinformation thrives in situations with overt or latent conflict that feature rigid social structures and hostile belief systems against those outside of the in-group [73]. False information about rival groups is more likely to be endorsed – believed and shared – by those who perceive conflict levels to be high [74]. For example, a study of conflict-affected regions of Southeast Asia showed that people were more likely to believe false content that was shared repeatedly, confirmed their existing worldviews, and played on their security-related anxieties [75].

Research has shown that disinformation campaigns often target civilian populations rather than specific political adversaries [76], and states are suggested as being more likely to lie to their own domestic public than abroad [77]. Disinformation campaigns are wielded by a scope of actors, not limited to state leaders and apparatus, but also rebel groups, terrorist groups, and members of the public. Disinformation creates the potential for actors to engage in conflict in new ways and to overcome barriers to entry for traditional combat, such as physical distance and fighting capacities [78]. Using deep fakes and other artificial intelligence technologies [68] can doubly serve as a new source of income – with the potential to be used to fund other additional violence – when sensationalist content drives pay-per-click traffic and user engagement [79]. Disinformation campaigns may make it possible to fight while avoiding open armed attacks on the public that are costly and prohibited under International Humanitarian Law (IHL) [80]. The International Committee of the Red Cross does not consider hybrid threats and warfare, including disinformation specifically, with novel legal criteria, but uses long-established legal definitions to consider how disinformation may be wielded as a tool in international and non-international conflict [81].

Some political objectives of disinformation depend on lies being believed or accepted and acted upon by the public. Disinformation can sway people to directly engage in or condone coordinated political violence [78,82]. Further implications of the spread of disinformation can result in the dehumanisation of political adversaries or aggressors, and in doing so encourage people to dissolve horizontal social ties that otherwise prevent large-scale violence [83]. For example, channels of disinformation precipitated via media communications and were used throughout the genocide in Rwanda in 1994 to incite large-scale public participation and in its aftermath to deny that the genocide happened [84,85]. Additionally, political campaigns spreading falsities can influence populations to harm themselves, through distorting information with the intention to impede people from meeting their basic needs as well as deliberately harm the mental health of exposed populations [76].

While much wartime propaganda is aimed at creating and defending a specific narrative of identity, experience, or order, la Cour [67, p.711] argues that it is more likely that, ‘disinformation has a destructive aim to disrupt, divide and confuse.’ The goal of disinformation, therefore, can also be to disintegrate cohesive narratives rather than create new ones. In international warfare, disinformation campaigns help actors to prepare for war by creating moral ambiguity, which was seen leading up and throughout the ongoing Russian invasion of Ukraine [80]. Disinformation has been aimed at undermining trust and legitimacy in political systems and institutions, including by attacking the ‘mainstream media’ that is intended to serve as a watchdog in liberal democracies. Moreover, ‘new wars,’ where the objective is to achieve political rather than territorial control, may be advanced through a steady stream of disinformation [86].

### Disinformation and public health violence

This section provides a review of the literature on conflict and disease outbreaks, including the role of disinformation. Conflict-affected contexts are not only likely to feature various forms of disinformation, but they are also often places of infectious disease emergence and re-emergence [87,88]. Armed conflict is a risk factor for outbreaks of several infectious diseases, including cholera [89], measles [90], meningitis [91], polio [92], cutaneous leishmaniasis [93], and Ebola [94].

Armed conflict is a risk factor for disease outbreaks in part due to reduced healthcare access from limited facilities and medical personnel. For example, medical personnel and healthcare facilities were targeted during Syria’s civil war, with 782 medical personnel killed and 492 medical facilities attacked between 2011 and 2017 [95]. Similarly, in the Gaza Strip from 7 October to 22 November 2023, over 60% of health facilities were structurally damaged and over 35% functionally destroyed by the Israeli military [96]. Polio vaccine workers in Pakistan and Afghanistan are routinely attacked and killed, with Wahid et al. [97] documenting these types of attacks since 2012. Years of conflict and instability can lead to a further breakdown in infrastructure, such as water and sanitation, healthcare, and housing; an increase in poverty due to a lack of ability to generate income and access education; lack of supplies and a collapse of health systems; the inability of healthcare providers and populations to reach each other; and an increase in mistrust in the government, non-governmental organisations, and healthcare professionals and providers [98,99]. In such contexts, basic healthcare and treatments to maintain health and prevent the spread of disease are curtailed.

Disinformation plays a role in limiting access to healthcare even when it is available. For example, the Central Intelligence Agency (CIA) of the United States developed a fake hepatitis B vaccination program to obtain DNA that helped to track down and kill Osama bin Laden in Pakistan. When this story was released, it fuelled unfortunate but somewhat cogent existing hesitancy about polio vaccines in Pakistan [100] with disinformation propagated in-person and online by armed militants and insurgency supporters [17]. The Pakistani doctor who assisted the CIA was jailed in Pakistan for treason, and polio continues to be endemic in Pakistan and Afghanistan. This experience left a long shadow and now supports the continuation of polio’s infectious disease outbreak [101].

Militarisation of healthcare centres can additionally stoke a lack of trust in healthcare services, and a fine balance is needed between increased security to protect staff and patients but not too much to increase suspicion that outbreaks or treatments are politically connected [102]. This may be difficult but not impossible to achieve. A successful example includes the Kaga Bandoro hospital in the Central African Republic that organised a community cash-for-work program to build a perimeter fence with clear medical signage alongside conducting awareness-raising sessions for community members including armed actors. This community-based initiative improved security not only due to the physical structure but also by fostering community protection mechanisms and contributing to household income for the 160 local people employed in the project [103].

While disease outbreaks do not cause conflicts, disinformation may reinforce ongoing division and violence. For the COVID-19 pandemic, disinformation around its origins and spread were associated with increased hate crimes against Asian Americans in the United States [104] and exacerbated ethnic stigmatisation of the Roma in Romania [105]. As such, an academic publication argued that disinformation itself should be considered a form of biowarfare – in this case, for spreading unfounded information that microbes were manufactured as biological weapons to advance political aims [106]. In some cases, the effects of disinformation on public health violence have lingered beyond the pandemic years. Disinformation about the COVID-19 vaccines prompted an armed gunman to open fire on the Centers for Disease Control (CDC) in the US in 2025, killing an officer before killing himself [107].

Yet, other research has shown that the COVID-19 pandemic was followed by de-escalation of armed conflict in certain cases, irrespective of the global reach of disinformation [108]. This study demonstrates that the relationship between public health disinformation and conflicts is not causal or unavoidable. This highlights the potential for governance not only to avoid the negative impacts of disinformation on conflict but also to leverage pandemics and other public health crises for improved global and public health cooperation.

## Discussion

This review demonstrated that disinformation has long been used as a tool to advance political goals including conflict and violence, and actors also leverage disinformation to disrupt and destabilise systems, with public health as one of the most relevant arenas. This review suggests a mutually influencing relationship between conflict and public health mainly owing to indirect mechanisms that limit access to quality healthcare even when it is otherwise available through discouraging use of services and uptake of guidance. Conflict-affected areas thus feature opportune conditions for disease outbreaks to take hold in their populations, and disease outbreaks go on further to strain sociopolitical relations and the institutional capacity of already-fragile systems surrounding public health.

Public health disinformation serves as a lynchpin in this self-reinforcing relationship. Disinformation and conflict are already widely considered major global and public health problems [109,110], but this research suggests that they are an interconnected challenge. Public health disinformation has encouraged attacks on healthcare workers and facilities as well as members of civil society and the general public, and it has also served to confuse and disorient people about recommended health behaviours and best practices during disease outbreaks. In doing so, public health disinformation has undermined trust in global and public health authorities to provide accurate information and adequate services.

### Diminishing global and public health governance

The mutually influencing and reinforcing relationships between public health disinformation, conflict, and disease outbreaks are met with weakened global and public health governance. Multilateral cooperation for global health is experiencing a downturn that is making the world even less prepared to manage future global health challenges including outbreaks despite ever-improving technologies [111].

Experiences during the COVID-19 pandemic have prompted the global health community to fight against public health disinformation by cracking down on social media platforms, increasing public awareness, and improving media literacy, especially focused on disinformation generated and propagated by artificial intelligence [112]. Not only do these strategies not engage with the political or social processes driving disinformation and its impacts, but they have also been met with limited uptake. For example, popular podcasting platforms, like Spotify and Apple, and social media platforms, like X (formerly Twitter) and Facebook, which are used by information actors ranging from politicians to influencers, have rolled back already-limited policies to detect false information, including third-party fact-checking [113,114]. Video venues, notably YouTube and TikTok, also have huge user numbers with limited fact-checking or information vetting. These strategies assume that disinformation is only generated by ‘bad actors’ and disseminated naively by users, but people share and do not challenge false information not only because they believe it but also knowingly due to a range of motivating factors [115,116].

Meanwhile, failures of public health information authorities have been increasingly brought to light. At the global institutional level, the WHO is far from infallible; it has been called out for disseminating information recommending potentially ineffective and harmful psychiatric drugs [117]. Governments and public health authorities spread false information at times, including instances where authorities deliberately suppress the truth and label it as false, and those with authority and power often enjoy impunity for spreading disinformation [118]. For example, during the COVID-19 pandemic, whistleblowers around the world challenged government control and suppression of information, but often faced censorship and disciplinary measures [119]. One of the most prominent instances was Dr. Li Wenliang’s warning about a new coronavirus outbreak in Wuhan, China, in December 2019, which resulted in him being summoned and reprimanded by the police [120]. After being publicly humiliated for spreading ‘lies’ which turned out to be true, he eventually died of the disease [121].

International institutions like the United Nations including the WHO have little capacity to set, monitor, and enforce supranational binding rules and they were seen to be in an overall state of decline long before 2025’s substantive change in the world development landscape [122,123]. Global and public health governance including but not limited to the WHO has been critiqued as ‘de-democratised’ and failing to engage with domestic socio-political power relations [124]. Argentina announced its departure from the WHO due to ‘deep differences’ with the management of health issues and ‘a lack of independence from the political influence of other states’ [125, p.3–4). In 2020, then-Brazilian President Jair Bolsonaro announced the potential to leave the WHO after the COVID-19 pandemic due to his critique of it being a ‘partisan political organization’ [126, p.2), and may have gone through with it had he been reelected. Whether these reflect the true or comprehensive reasons for leaving the WHO, they can be situated within broader political trends. The International Committee of the Red Cross (ICRC) [81, p.1360] described ‘a shift in the narrative of international relations: globalization and multilateralism, once dominant themes, are being replaced by a story that emphasizes competition over cooperation and conflict preparedness over peace.’

Journalism has been suggested as the ‘fourth estate’ that has the essential function of monitoring governments and those with power and keeping the public informed [127], and which could play a powerful role as a watchdog for public health dis/information. However, freedom of the press globally ‘now stands at an unprecedented, critical low as its decline continued in 2025’ with trends seen across regions and polities [128, p.1). This is despite popular narratives that emphasise freedom of speech over combatting disinformation. The increasing prosecution of whistleblowers is seen in the US, for example, with President Barack Obama prosecuting more whistleblowers than all previous administrations before him combined [118,129], despite the Administration proclaiming an agenda for ‘unprecedented’ government transparency and openness [130]. Moreover, traditional news media is becoming increasingly less relevant for producing news content, with young audiences especially being exposed to news through social media and television [131].

### Leveraging and strengthening health diplomacy

With these trends and challenges in mind, it is far from clear who has the legitimate authority not only to produce ‘true’ information but also to label competing narratives as ‘false.’ The desire for an overarching information authority may be misguided, as authority lies among and between entities of different types and at different levels that serve their subjects [132]. This section conducts conceptual theorising based on the integrative narrative review to consider how health diplomacy could be leveraged as a tool to weaken the drivers and impacts of global and public health disinformation while also strengthening cooperative governance amidst conflict.

The field of global health is fundamentally built on cooperation – across political boundaries and technical areas of expertise – to achieve common goals, and the field of health diplomacy seeks to strengthen and enhance this cooperation. Health diplomacy ‘represents an important forum for negotiations on global policy issues that shape and influence the global environment for health. It brings together a wide range of actors in areas that affect public health’ [133, p.2). Falling under the umbrella of health diplomacy are efforts to improve the coordination of technical expertise and authority structures, as well as those aimed to build relationships and fair health outcomes across political and jurisdictional boundaries [134–136]. Practices of health diplomacy have led to past successes, for example the global eradication of smallpox in 1979, even in an era of significant global tension [137]. Further key examples of health diplomacy across and between countries include the global eradication of Rinderpest in 2011 [138] and the launching of the *Global Polio Eradication Initiative* (GPEI) in 1988.

Global and public health diplomacy have already made strides towards facilitating information sharing and relationships between states and technical actors. Essential to global and public health governance is not that it is always right as an authority, but that its processes encourage open dialogue, it can correct its own errors, and it can organise itself transparently. Rather than double down on the strength of a single global health authority producing a single narrative, global actors may choose to facilitate information production and sharing that is more transparent to the public. In doing so, they may highlight the multiple streams of information produced by diverse actors, including state-based and regional actors that can take more ‘courageous’ and normative stances on public health concerns outside of political institutions that paint themselves as neutral and objective [139]. Moving away from singular messaging produced through a consensus process that is swayed by political power, health diplomacy could instead open pathways towards more transparency with the public on discourse, including disagreements and discrepancies.

Whereas health diplomacy has conventionally focused on improved state to state relations, health diplomacy can adopt multitrack strategies that include civil society in diplomacy. Multitrack diplomacy could aim to harness conflict more constructively and produce healthy debates in civil society to reignite the social creation of public health information. Agonistic dialogue is a feature of deliberative democracy that engages conflict dynamics and encourages discussion among parties as legitimate political opponents rather than forcing consensus and suppressing dissent [140–142]. For global and public health, this may involve integrating multiple and localised sources of data into global and public health information. This process may benefit from being linked to statistical data and through developing relatable and contrasting storylines at individual and social scales through localised and participatory processes [143] that also incorporate elements of ‘infotainment’, possibilities, and action [131]. Storylines can be co-produced in ways that are sensitive to power differences and divides, and which communicate not only risks but also aspirations. When people’s interests are already met through information that is meaningful and relevant to them, they may be less inclined to turn to sources that purposefully manufacture lies.

Health diplomats must also be willing to have difficult conversations, including around how health systems have created and perpetuated harms. Public health distrust develops over time and is perpetuated by systems of injustice, and trust is not just built through airing injustices but also through growing away from them [144]. Best practices suggest that dialogue should be accompanied by safeguarding against deceptive health interventions and other injustices [145,146]. Restorative justice practices offer a framework to address and discuss past harms, including providing opportunities for perpetrators and victims to ask questions and agree on how to redress harms, prevent future harms, and repair their relationship [147]. However, optimism must be tempered with evidence showing that attempts to rebuild institutional trust can initially undermine it [148]. For example, the CIA’s pledge not to conduct additional fake vaccination campaigns roused concerns that resurfacing past experiences may reinvigorate local fears in current programs against polio in Pakistan [149].

Health diplomacy should also engage the media and private sectors to find common ground. Podcasting and social media platforms play an important role in identifying false information and ensuring tougher community guidelines, including people claiming credentials they do not have and citing data that has proven to be incorrect. However, rather than swiftly remove such posts and/or profiles, it may be instructive to post disproven information on a dedicated page along with the evidence and arguments against it to increase awareness for media consumers and empower them to come to their own informed conclusions for their health. Further, efforts could be made to engage social media influencers to communicate along these lines, and punish those for persistent offences [150], since they already serve as a source of health information for young adults [151]. It may be advisable to encourage more active presence of public health experts and agencies on social media [150,152], including campaigns partnering with social media influencers and health professionals. One successful example is Jo’s Cervical Cancer Trust that collaborated with British social media influencer Zoe Sugg on an awareness campaign in 2019 [150].

In so doing, health diplomacy could be leveraged to build *mis*trust rather than unquestioning trust. Healthy mistrust is a feature of civic vigilance and critical engagement, and may paradoxically improve people’s ability to discern and understand different accounts of reality [153,154]. This may be especially important given that both disinformation and new technologies are likely to be mainstays in global and public health. Health education and literacy have been recentered on the public health agenda especially in the wake of the COVID-19 pandemic [155], including through increasing access to evidence-based health information and fostering critical thinking capacities among communities to recognise and mitigate public health disinformation campaigns and their potentially destabilising impacts on health and health systems. Health diplomacy could additionally foster respectful debate and mutual learning from multiple perspectives and lived experiences rather than asserting truth versus lies for global and public health.

## Conclusions

As highlighted by the WHO [156, p.1), within a digitised era after the COVID-19 pandemic, ‘health challenges cannot be resolved at solely the technical level – they require political negotiations and solutions, and often need to involve a wide range of actors.’ Naturally, this baseline is naive since health challenges throughout human history have never been only technical, with political and social forces always determining public health outcomes. Misinformation and disinformation predating the COVID-19 pandemic – including smallpox, cholera, the 1918–1920 influenza pandemic, and HIV [157] – starkly illustrate that these challenges are not new. As the world is witnessing the deterioration of the WHO and other international institutions as global authorities, now is a salient time for the global and public health community to rethink its politics of engagement.

The real challenge for pandemic preparedness may be to foster the political will to steer through a crisis of legitimacy not through attacks but through (re)connection and cooperation. Whereas global and public health diplomacy has historically focused on the high politics of states, this research conceptually theorised on how global and public health diplomacy could be integrated with other strategies to meet the intertwined challenges of disinformation and conflict for disease outbreaks. In line with fundamental understandings of conflict as natural and even having the potential to be constructive and lead to improved outcomes [28], an important function of global and public health governance is to provide mechanisms for managing these conflicts. Based on this integrative narrative review and conceptual theorising, we make the following actionable recommendations for health diplomacy:

1. Health diplomacy should seek to strengthen global and public health governance not on the grounds of their ability to produce truth or consensus, but based on their capacity for open, fruitful discussions and transparent sharing of information and knowledges.
2. The global and public health community should expand multitrack health diplomacy to engage elected and appointed officials, civil society organisations, traditional and new media outlets, local leaders, and members of the public in cooperative governance;
3. Health diplomats should expand health literacy programming to help people better understand disinformation and how it impacts them, their families and communities, and their broader societies, with an emphasis on what people can do to prevent and limit the impacts of public health disinformation;
4. Health diplomacy should invest in multi-directional communication strategies, including engaging with social media influencers and public figures, not only to disseminate directives but also to engage in constructive debates and discussions;

This call to action is not a quick fix: it is governed by dynamics that are much slower than those of disinformation, and it depends on even longer-term global and public health governance reform. As such, investments in technical and technological countermeasures to combat disinformation in real-time, including restoring rules for social media platform monitoring and fact-checking processes, should be made, but as complementary to health diplomacy rather than representing the central effort. The limitations of this research as an integrative narrative review with conceptual theorising include that the findings are more subjective; they are informed by the authors’ areas of expertise; and the applied limitations include lack of funding and political will to reform global and public health governance and to invest in health diplomacy.

The COVID-19 pandemic reminded the world of the profound interconnectedness of global and public health and the necessity to build trust across governments, populations, and systems to achieve common goals. Contending with multiple strands of information, including those that are fabricated and falsified, should be considered a fixture for global and public health. A more transparent and inclusive public health information ecosystem fostered through health diplomacy offers a long-term pathway to mitigate the harms of disinformation and strengthen preparedness for future global and public health crises.
